# Abdominal obesity exhibits distinct effect on inflammatory and anti-inflammatory proteins in apparently healthy Japanese men

**DOI:** 10.1186/1475-2840-6-27

**Published:** 2007-10-01

**Authors:** Makoto Nishida, Toshiki Moriyama, Yoshiro Sugita, Keiko Yamauchi-Takihara

**Affiliations:** 1 Health Care Center, Osaka University, Machikaneyama, Toyonaka, Osaka 560-0043, Japan

## Abstract

**Background:**

Since visceral fat tissue is known to release various inflammatory and anti-inflammatory cytokines, abdominal obesity may play a key role in the inflammation associated with metabolic syndrome (MetS). However, few studies have determined precise relationships of abdominal obesity with inflammatory markers in MetS. To clarify the importance of abdominal obesity in sub-clinical inflammation, we examined the changes of inflammatory markers in clustering of MetS components with or without abdominal obesity.

**Methods:**

Subjects consisted of 326 apparently healthy Japanese men (age: 30 to 59 years) who underwent health examination in the Osaka University Health Care Center. MetS components were assessed and serum levels of high sensitive C-reactive protein (hs-CRP), interleukin (IL)-6 and adiponectin were examined in all subjects.

**Results:**

Subjects with abdominal obesity (waist circumference ≥ 85 cm) showed higher serum hs-CRP and IL-6 levels and a lower adiponectin level than those without abdominal obesity. Serum levels of hs-CRP and IL-6 significantly increased in association with clustering of MetS components in the subjects with abdominal obesity, but not in those without abdominal obesity. On the other hand, serum adiponectin level exhibited a little change with clustering of MetS components in the subjects with abdominal obesity. Significant negative correlation between adiponectin and hs-CRP was observed in the subjects with abdominal obesity, however this correlation was not detected in obese subjects defined by body mass index ≥ 25.

**Conclusion:**

Inflammatory status is not exaggerated by clustering of MetS components in the subjects without abdominal obesity. Abdominal obesity may exhibit distinct effect on inflammatory and anti-inflammatory proteins and modulate inflammatory network in MetS.

## Background

National Cholesterol Education Program (NCEP)-Adult Treatment Panel (ATP) III, and American Heart Association (AHA)/National Heart Lung and Blood Institute (NHLBI) proposed definitions of the metabolic syndrome (MetS) as a cluster of at least 3 of 5 following risk factors, abdominal obesity, raised blood pressure, hyper-triglyceridemia, hypo-HDL-cholesterolemia, and raised fasting blood glucose [1,2]. The primary mechanism for clustering of metabolic abnormalities has yet to be elucidated, but insulin resistance or accumulation of visceral fat is believed as an encouraging candidate [3,4]. As for visceral fat, the International Diabetes Federation (IDF) newly recommended a definition in which abdominal obesity is the essential component of MetS [5,6].

Recent studies have proposed an association between MetS and inflammatory markers [7-10]. Abdominal obesity, especially a visceral fat, may play a key role in these situations, because adipose tissue is known to secrete many bioactive substances, including inflammatory and anti-inflammatory proteins [11,12]. However, only few studies have precisely examined the significance of abdominal obesity in the inflammatory changes observed in MetS [13,14].

In the present study, we examined inflammatory and anti-inflammatory proteins in Japanese middle-aged men, and investigated whether presence of abdominal obesity has an impact on the inflammatory responses. We here describe unique associations among abdominal obesity, clustering of MetS components, and inflammatory and anti-inflammatory markers.

## Methods

### Study population

A total of 326 apparently healthy Japanese men, 30 to 59 years of age, who underwent health examination in the Osaka University Health Care Center, were evaluated with MetS components. Individuals with a history of acute illness within 2 weeks or who had taken any medicine were excluded (n = 31) in order to avoid effects on serum levels of inflammatory proteins. Same numbers of subjects with or without abdominal obesity were consecutively selected. Comparison was performed between an "abdominal obesity group" who met the criterion of waist circumference for abdominal obesity and a "non-abdominal obesity group" who did not meet this criterion. Informed consent was obtained from all subjects prior to participation in the study following approval of the study by the Ethics Committee of Osaka University.

### Risk factor assessment

Waist circumference at the umbilical level was measured in the late exhalation phase in standing position. Abdominal obesity was diagnosed by waist circumference ≥ 85 cm using the guidelines for abdominal obesity in Japanese individuals [15]. To examine the significant role of abdominal obesity, we employed the definition by IDF [5]. In the criteria of IDF, abdominal obesity is a prerequisite risk factor, and MetS was defined by the existence of abdominal obesity and at least 2 of 4 MetS components defined as follows: 1)    hypertriglyceridemia: a serum level of triglycerides (TG) ≥150 mg/dl; 2) low HDL cholesterolemia: a serum level of HDL cholesterol (HDL-C) < 40 mg/dl; 3) hypertension: systolic blood pressure (SBP) ≥ 130 mmHg, or diastolic blood pressure (DBP) ≥ 85 mmHg; 4) high fasting glucose: plasma level of glucose (FPG) ≥100 mg/dl.

### Laboratory measurements

Serum was collected from subjects after an overnight fast and kept at ≤ -20°C until assay. Serum immunoreactive insulin was measured by a standard radioimmunoassay method (Dinabott Co., Tokyo, Japan). Homeostasis model assessment of insulin resistance (HOMA-IR) was defined as the product of fasting plasma insulin (μU/ml) and glucose (mg/dl) divided by 405 [16]. Low density lipoprotein cholesterol (LDL-C) was calculated by the Friedewald formula. However, LDL-C was not calculated if the TG level was >300 mg/dl. Serum high sensitive-C reactive protein (hs-CRP) level was measured with an immunoenzymeassay (Dase Behring, Marburg, Germany). Serum interleukin (IL)-6 concentration was measured in duplicate with a chemiluminescent enzyme immunoassay (CLEIA) (Fujirebio Inc., Tokyo, Japan), and adiponectin was measured duplicate by a sandwich enzyme-linked immunosorbent assay (ELISA) system by adiponectin ELISA kit (Otsuka Pharmaceutical Co., Tokushima, Japan) [17,18].

Before conducting present study, interclass CVs of hs-CRP, IL-6, and adiponectin measurements were examined (n = 40). Mean CVs of hs-CRP, IL-6, and adiponectin measurements in our assays were 1.1%, 4.5%, and 1.2%, respectively. The same lots of these kits were used in the study to keep reliability of the measurements.

### Statistical analysis

All values are mean ± SD. Pearson's correlation coefficients were calculated among skewed variables after logarithmic transformation of variables. ANOVA with Bonferroni post hoc test was used to assess difference between groups. Values of P < 0.05 were considered statistically significant.

## Results

Profiles of the study subjects are shown in Table 1. Traditional risk factors were exacerbated and HOMA-IR was increased in abdominal obesity group as compared with non-abdominal obesity group. Abdominal obesity group presented significantly higher hs-CRP and IL-6 levels, and a lower adiponectin level than non-abdominal obesity group.

**Table 1:** Characteristics of the study subjects

	All Subjects	Non-abdominal obesity group	Abdominal obesity group	*P*
n	326	163	163	
Age, y	45.1 ± 8.1	45.3 ± 8.4	44.8 ± 7.7	n.s.
Waist, cm	84.8 ± 8.2	78.3 ± 4.7	91.2 ± 5.2	<0.0001
BMI, kg/m^2^	24.3 ± 3.3	22.1 ± 2.0	26.6 ± 2.8	<0.0001
SBP, mmHg	123 ± 16	120 ± 14	127 ± 17	<0.0001
DBP, mmHg	79 ± 12	76 ± 11	82 ± 13	<0.0001
TC, mg/dl	208 ± 35	203 ± 33	214 ± 37	0.003
TG, mg/dl	131 ± 128	100 ± 53	162 ± 168	<0.0001
HDL-C, mg/dl	58 ± 14	62 ± 15	53 ± 11	<0.0001
LDL-C, mg/dl	125 ± 34	120 ± 31	129 ± 37	0.03
FPG, mg/dl	91 ± 10	89 ± 9	93 ± 10	0.0009
HOMA-IR	1.35 ± 2.03	0.75 ± 0.29	1.87 ± 2.67	0.03
hs-CRP, mg/L	0.68 ± 0.77	0.58 ± 0.82	0.78 ± 0.71	0.018
IL-6, pg/ml	1.26 ± 0.75	1.18 ± 0.66	1.34 ± 0.83	0.045
Adiponectin, μg/ml	6.3 ± 2.9	7.1 ± 3.3	5.5 ± 2.1	< 0.0001

Data are mean ± SD. Abdominal obesity and non-abdominal obesity groups are the subjects who met or did not meet the waist criteria (≥ 85 cm). BMI: body mass index; SBP: systolic blood pressure; DBP: diastolic blood pressure; TC: total cholesterol; TG: triglycerides; HDL-C: HDL cholesterol; LDL-C: LDL cholesterol; FPG: fasting plasma glucose; HOMA-IR: homeostasis model assessment of insulin resistance; hs-CRP: high sensitive C reactive protein; IL-6: interleukin-6. Statistical significance was examined between non-abdominal obesity and abdominal obesity groups.

**Figure 1:**
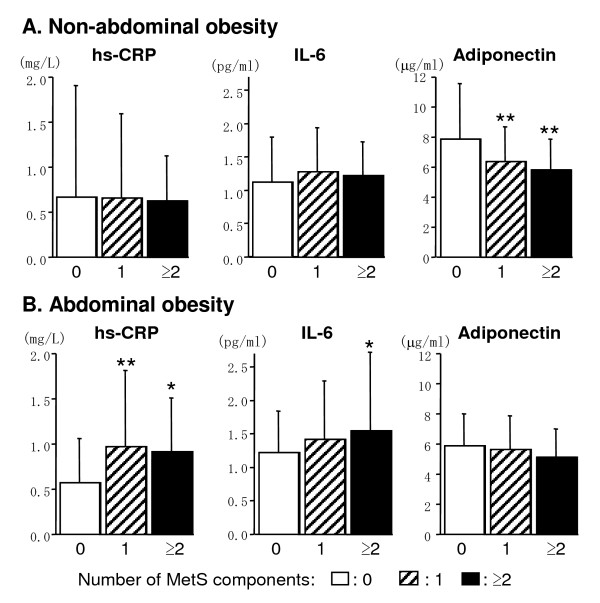
**Serum levels of hs-CRP, IL-6, and adiponectin**. **A**. Serum levels of hs-CRP, IL-6, and adiponectin in non-abdominal obesity group. Open, hatched, and closed columns represent the subjects with no, one, and two or more metabolic syndrome components except for waist criteria, respectively. Numbers of subjects in open, hatched, and closed columns were 95, 50, and 18, respectively. **B**. Serum levels of hs-CRP, IL-6, and adiponectin in abdominal obesity group. Columns represent same as described in **A**. Numbers of subjects in open, hatched, and closed columns were 57, 57, and 49, respectively. Data are mean ± SD. *p < 0.05, **p < 0.01 vs. no metabolic syndrome component (open columns). hs-CRP: high sensitive C-reactive protein; IL-6: interleukin-6; MetS: metabolic syndrome.

The effects of clustering MetS components on inflammatory status were compared between these two groups. Interestingly, in non-abdominal obesity group, serum levels of hs-CRP and IL-6 changed a little in association with clustering of MetS components (Figure 1A). However, serum levels of hs-CRP and IL-6 significantly increased in association with clustering of MetS components in abdominal obesity group (Figure 1B). On the other hand, serum adiponectin level exhibited a little change with clustering of MetS components in abdominal obesity group (Figure 1B), while it was significantly decreased in non-abdominal obesity group. These findings suggest that abdominal obesity may be a key factor in modulating the inflammatory reactions observed in MetS.

We next examined whether these changes of inflammatory markers associate with traditional risk factors. The mean value of risk factor in the subjects either with low or high level of inflammatory marker was compared. Significance of difference was summarized in Table 2. The mean values of waist, TG, and HDL-C showed significant changes associated with classification of adiponectin and hs-CRP levels. FPG was significantly changed between subjects with high and low adiponectin levels. SBP, and DBP levels were significantly changed between subjects with high and low hs-CRP levels. Moreover, stepwise regression analysis revealed waist circumference was a significant determinant for the levels of adiponectin, IL-6, and hs-CRP (Standard regression co-efficient: -0.237, 0.115, and 0.256; F value: 18.6, 4.1, and 20.9; respectively)

**Table 2:** Difference in mean values of risk factors between the subjects with *"High" *and *"Low" *inflammatory markers

	*"High" and "Low" Groups*		
	
	Adiponectin	IL-6	hs-CRP
Waist	**<0.0001**	0.18	**<0.0001**
SBP	0.66	0.069	**0.0003**
DBP	0.21	0.40	**0.002**
LDL-C	0.82	0.21	0.20
TG	**0.0031**	0.10	**0.0078**
HDL-C	**<0.0001**	0.098	**<0.0001**
FPG	**0.019**	0.91	0.46
HbA1c	0.19	0.62	0.50
HOMA-IR	0.11	0.30	0.076

Groups of *"High" *and *"Low" *inflammatory markers were divided by the half number of the subjects. Abbreviations are same as described in Table 1. **Bold **values indicate p < 0.05.

Networks of inflammatory and anti-inflammatory proteins may be altered by the presence of abdominal obesity. Therefore, we next examined the correlation of these markers both in the subjects with abdominal obesity and in obese subjects defined by body mass index (BMI) ≥ 25. As shown in Table 3, correlation was not observed between serum adiponectin and hs-CRP levels in obese subjects (BMI ≥ 25), and even in those with two or more MetS components. However, there was a significant negative correlation between serum adiponectin and hs-CRP levels in abdominal obesity group (r = -0.23 [95%CI, -0.37 to -0.08]). In addition, significant correlation was detected in the subjects with abdominal obesity with two or more MetS components, i.e. MetS (r = -0.29 [95%CI, -0.53 to -0.01]). No significant correlation was observed between adiponectin and IL-6 in both obese and abdominal obese subjects (data not shown).

**Table 3:** Correlation between serum adiponectin and hs-CRP levels in obesity and abdominal obesity groups

Group	n	Correlation coefficients	95% CI	*P*
Obesity (BMI ≥ 25)	129	-0.05	-0.22 to 0.13	0.61
with ≥ 2 MetS components	42	-0.12	-0.41 to 0.19	0.44
Abdominal obesity (Waist ≥ 85)	163	**-0.23**	**-0.37 to -0.08**	**0.003**
with ≥ 2 MetS components	49	**-0.29**	**-0.53 to -0.01**	**0.045**

Obesity group includes the subjects with body mass index (BMI) ≥ 25. Abdominal obesity group includes the subjects with waist circumference ≥ 85 cm. MetS: metabolic syndrome. Significant negative correlation between adiponectin and hs-CRP was detected only in the abdominal obesity group. **Bold **values indicate p < 0.05.

## Discussion

It is considered that clustering of metabolic abnormalities presents synergistic effects on cardiovascular complications beyond the sum of effects of individual abnormalities [19]. Therefore, to elucidate the mechanism in clustering of metabolic abnormalities is an important issue. In the present study, percentage of the subjects with two or more MetS components was markedly higher in the abdominal obesity group (30.1%) than the non-abdominal obesity group (11.0%) (data not shown). Abdominal obesity, therefore, might play a pivotal role among various MetS components in the practical definition of MetS. When abdominal obesity was absent, inflammatory proteins, such as hs-CRP and IL-6, did not increase significantly with clustering of MetS components (Figure 1A), despite the fact that previous studies found an association between CRP and MetS [7,20]. Our results clearly demonstrated association between hs-CRP/IL-6 levels and clustering of MetS components predominantly in the presence of abdominal obesity. Although BMI might also alter inflammatory status, classification by BMI did not present clear association between inflammation and clustering of MetS components (data not shown).

Inflammation may be an important underlying etiology of MetS [8] through its various effects mediated by adipose tissue-derived factors, such as leptin, adiponectin, IL-6, IL-10 and etc. [12]. Many studies demonstrated associations between inflammatory proteins and MetS, abdominal obesity, or insulin resistance. Our results also demonstrate the importance of waist circumference in the changes of inflammatory markers (Table 2). However, abdominal obesity has usually been evaluated as one of the components of MetS. In the present study, we clearly showed that abdominal obesity exhibits distinct association with inflammatory and anti-inflammatory proteins in clustering of MetS components in apparently healthy subjects.

Adiponectin, an anti-inflammatory protein [21], has been demonstrated to be insulin-sensitizing and anti-atherogenic factor, and is considered a key component of MetS [11,22,23]. Serum adiponectin level was significantly lower in the subjects with abdominal obesity than those without it, and a marked decrease was observed with clustering of MetS components only in the subjects without abdominal obesity. In agreement with previous reports, low level of serum adiponectin is a potent risk for MetS, and elevation of serum hs-CRP and IL-6 concentration enhances the incidence of MetS in the subjects with abdominal obesity.

Previously, an inverse correlation between expression levels of CRP and adiponectin in adipose tissue from the patients with coronary artery disease had been demonstrated [24]. In the present study, a significant negative correlation was observed between serum adiponectin and hs-CRP levels in the subjects with abdominal obesity, but not in obese subjects. These results suggest that abdominal obesity may exhibit distinct effect on inflammatory and anti-inflammatory proteins. Recently, it is reported that portal concentration of IL-6 is higher than peripheral concentration of it, and visceral fat is identified as an important site for IL-6 secretion [25]. Thus, portal IL-6 from visceral fat might directly induce the production of CRP in the liver. In addition, macrophages infiltrating into visceral fat is another possibility for inflammatory networks specific to the abdominal obesity. It has been reported that macrophage is a source of adipose tissue-derived proteins [26]. The increased number of macrophages infiltrating the visceral fat tissue in individuals with abdominal obesity suggests that adipose tissue itself is a source and site of inflammation [27]. Thus, the inflammatory networks associated with abdominal obesity were distinct from those associated with mere "obesity". Després and Lemienx proposed visceral fat as a dysfunctional adipose tissue [4], and our results support this idea.

In conclusion, without abdominal obesity, inflammatory status is not exaggerated by clustering of MetS components in apparently healthy middle-aged men. Abdominal obesity may exhibit distinct effect on inflammatory and anti-inflammatory proteins and modulates inflammatory network in MetS.

## Competing interests

The author(s) declare that they have no competing interests.

## Authors' contributions

TM, YS and KYT conceived the study and participated in its design and coordination. MN performed the statistical analysis. MN and KYT drafted the manuscript and interpreted the data. All authors read and approved the final version of the manuscript.
